# The effects of linkage disequilibrium in large scale SNP datasets for MDR

**DOI:** 10.1186/1756-0381-4-11

**Published:** 2011-05-05

**Authors:** Benjamin J Grady, Eric S Torstenson, Marylyn D Ritchie

**Affiliations:** 1 Center for Human Genetics Research, Vanderbilt University Medical Center, Nashville, TN 37232, USA

## Abstract

**Background:**

In the analysis of large-scale genomic datasets, an important consideration is the power of analytical methods to identify accurate predictive models of disease. When trying to assess sensitivity from such analytical methods, a confounding factor up to this point has been the presence of linkage disequilibrium (LD). In this study, we examined the effect of LD on the sensitivity of the Multifactor Dimensionality Reduction (MDR) software package.

**Results:**

Four relative amounts of LD were simulated in multiple one- and two-locus scenarios for which the position of the functional SNP(s) within LD blocks varied. Simulated data was analyzed with MDR to determine the sensitivity of the method in different contexts, where the sensitivity of the method was gauged as the number of times out of 100 that the method identifies the correct one- or two-locus model as the best overall model. As the amount of LD increases, the sensitivity of MDR to detect the correct functional SNP drops but the sensitivity to detect the disease signal and find an indirect association increases.

**Conclusions:**

Higher levels of LD begin to confound the MDR algorithm and lead to a drop in sensitivity with respect to the identification of a direct association; it does not, however, affect the ability to detect indirect association. Careful examination of the solution models generated by MDR reveals that MDR can identify loci in the correct LD block; though it is not always the functional SNP. As such, the results of MDR analysis in datasets with LD should be carefully examined to consider the underlying LD structure of the dataset.

## Introduction

Linkage disequilibrium (LD) is defined as the nonrandom association of alleles at two or more loci [1]. The concept of LD and the statistics used to measure it relate directly to the frequency of ancestral recombination events which have separated the loci between which calculations are made. Frequent recombination between loci of genetic variation will result in linkage equilibrium. Thus it is often the case that when there is LD, it is due to physically linked genetic variants. LD can also result from population genetic events such as admixture and natural selection. GWAS take advantage of LD to be able to identify indirect associations but also suffer from strong LD over large genomic regions. The problem with LD in genomic data and its ability to confound analysis is illustrated by the human leukocyte antigen (HLA) locus, which was at the heart of several early spurious associations with susceptibility to immunological and infectious diseases as a result of 3 cM of high LD around the locus [2]. This example shows how long-range LD can confound analysis methods in their attempt to precisely identify loci associated with risk for disease.

**Figure 1:**
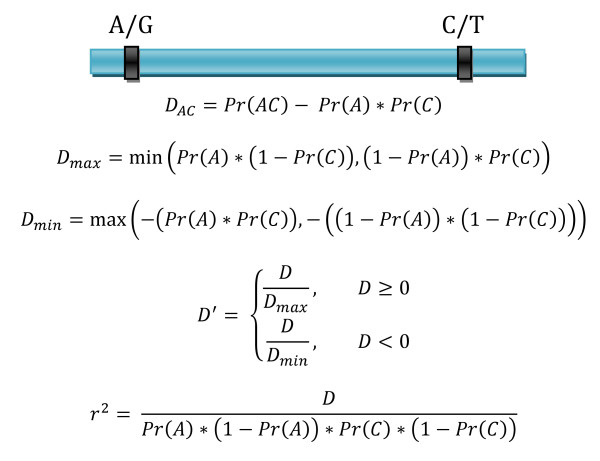
**Statistics used to measure LD**. Equations to calculate statistics commonly used to measure the degree of LD in genetic data.

While there are multiple statistics which can be used to measure the degree of LD between alleles at two genetic variants, the two which have become most popular in genetic epidemiology are r^2 ^and D'. Both statistics are based around D, the coefficient of disequilibrium. The value of D is the product of the frequencies of the alleles of interest at loci A and B subtracted from the frequency of chromosomes or gametes carrying both alleles (Figure 1) [1]. D' is the value of D divided by the absolute value of the maximum possible value D could take on given the allele frequencies and is thus a normalized statistic comparable between allelic pairs. The r^2 ^statistic is a correlation coefficient between the two alleles and will only be large if both are similar in frequency. For D, D' and r^2^, a value of zero is expected under the null hypothesis of no allelic association. While D can be negative or positive, both D' and r^2 ^range between zero and one. A value of one for D' indicates perfect disequilbrium as it relates to the absence of at least one of the expected haplotypes which would be possible given the alleles at the two loci. If r^2 ^takes the value of one, it means that the alleles at the two loci are perfectly correlated and are thus also in perfect disequilibrium. It is possible to have r^2 ^< 1 given D' = 1 but not the reciprocal. While the concept of linkage disequilibrium and the statistics used to describe it are specific to genetics, the phenomenon can more generally be considered as the presence of correlation between variables when thought of in regards to data mining and analysis methods.

**Figure 2:**
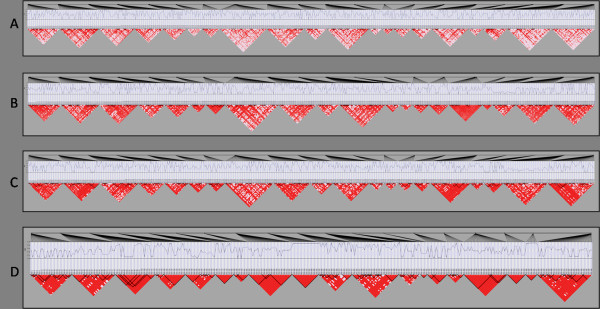
**Data pools of differing LD amounts**. The four different data pools used to generate data. A) 40% LD B) 60% LD C) 80% LD D) 95% LD.

**Figure 3:**
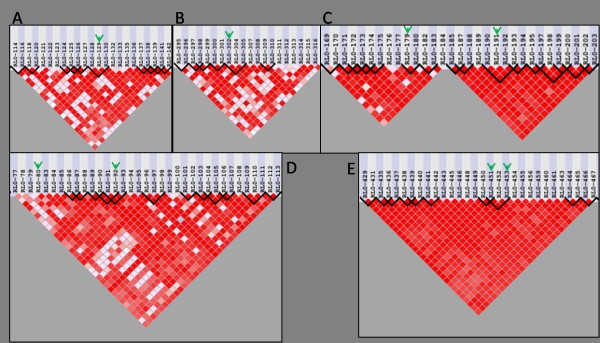
**Description of functional locus scenarios**. The five types of scenarios utilized in each population, where the green arrow indicates the disease susceptibility locus (or loci) and called LD blocks are outlined in black. A) One SNP in the middle of an LD block. B) One SNP in the center of a block of LD. C) Two SNPs in an epistatic interaction in separate LD blocks. D) Two interacting SNPs, one in a block of LD and one outside. E) Two interacting SNPs in the same block of LD.

Multifactor Dimensionality Reduction (MDR) is an analysis method designed to detect multi-locus interactions in large datasets. The MDR algorithm searches exhaustively among a set of categorical variables such as genotypes for interactions up to a specified order of degree (e.g. 2-way, 3-way). For each interaction model up to the specified level of complexity, each intersection of the potential values for the variables in the model is labeled as high risk or low risk based on the ratio of cases to controls who possess the intersection of values for the variables under examination (e.g. the AABB multi-locus genotype). The accuracy of this classification is then used as a metric of model importance, including the predictive value of the model in unseen data through the use of N-fold cross validation [3]. The design of the MDR algorithm renders it capable of detecting even those interactions for which the categorical variables have no detectable marginal effects. MDR has been used frequently in the field of genetic epidemiology - in the study of diseases such as Breast Cancer [3-5], Schizophrenia [6], and Type 2 Diabetes [7] - to search for interactions between single nucleotide polymorphisms (SNPs) implicating biological interactions of etiological significance. The performance of MDR has been examined in the presence of genetic heterogeneity, phenocopy and missing data but not in the presence of LD [8]. The goal of this study was to determine the sensitivity of MDR to detect the disease signal of functional loci in varying amounts of LD. Data ranging from low to high LD amounts were simulated using a forward-time genomic simulator (Figure 2). Cases and controls were subsequently drawn to by taking two chromosomes from a pool of simulated chromosomes and applying a penetrance function describing the probability of disease given the single- or multi-locus genotype present at the functional variant(s). The functional variants responsible for disease etiology were chosen to satisfy requirements of LD structure with surrounding SNPs and allele frequency (Figure 3). One hundred datasets with 1000 cases and 1000 controls were generated for each model. The resulting datasets were then analyzed with MDR and the sensitivity of the method was measured.

## Results

### Analysis of One-Locus Models

**Figure 4:**
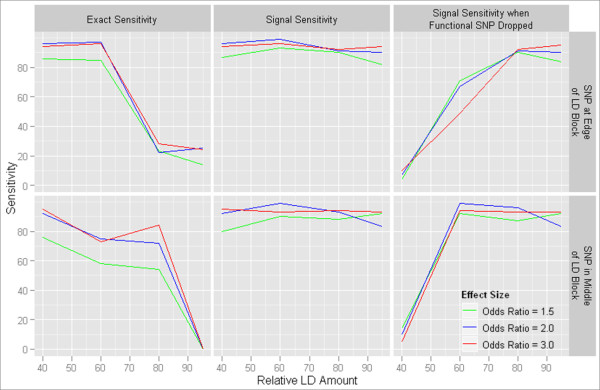
**Sensitivity of MDR for one-locus disease models**. The sensitivity of MDR to detect the functional one-locus model exactly, indirectly, and in the absence of the functional SNP when analyzing data with 40% LD, 60% LD, 80% LD or 95% LD and attempting to identify a signal in different positions of a block of LD.

Two single-locus models each with three effect sizes were simulated with genomeSIMLA and analyzed with MDR. Due to the presence of only small differences in sensitivity between the three effect sizes tested, the mean sensitivity across the effect sizes is used for comparison. The scenario in which the functional SNP is removed from the dataset prior to analysis was also examined. Results of these analyses are shown in Table 1. In all amounts of LD, the signal sensitivity did not differ largely between model types or amounts of LD and was usually above 90 for all effect sizes. The exact sensitivity, however, varied highly depending on the amount of LD. When 40% or 60% of the SNPs in the dataset were in high LD with at least one other SNP, the exact sensitivity was nearly equivalent to the signal sensitivity and was greater than 90. When there was a greater amount of LD, as in the 80% LD and 95% LD cases, the exact sensitivity dropped far below the signal sensitivity. The difference in exact sensitivities between the one-locus models also became more pronounced. In 80% LD, the signal sensitivity was 91 over the effect sizes for the case with a SNP at the edge of a block of LD and 91.7 when the SNP was in the middle of the block. The exact sensitivities for these same models were 24.3 and 70 respectively. For one-locus models in 95% LD, the signal sensitivity was 88.7 with a SNP at the edge of a block and 89.3 for a SNP in the middle of a block while the exact sensitivities were 21 and 0 respectively. The trends present in the one-locus models are illustrated in Figure 4. In general, the inaccuracies that detracted from the sensitivity scores in MDR were due to two-locus models being chosen in place of a one-locus model which was not counted towards detection sensitivity even if the functional locus was in this model.

**Table 1:** Sensitivity of MDR to detect one-locus disease models

Scenario	Relative LD	Effect Size	Exact Sensitivity	Signal Sensitivity	Signal Sensitivity when Functional SNP Dropped
SNP at Edge of LD Block	40%	1.5 OR	86	87	4
		
		2.0 OR	96	96	7
		
		3.0 OR	94	94	10
	
	60%	1.5 OR	85	93	71
		
		2.0 OR	97	99	67
		
		3.0 OR	96	96	49
	
	80%	1.5 OR	23	90	90
		
		2.0 OR	22	91	91
		
		3.0 OR	28	92	92
	
	95%	1.5 OR	14	82	84
		
		2.0 OR	25	90	90
		
		3.0 OR	24	94	95

SNP in Middle of LD Block	40%	1.5 OR	76	80	14
		
		2.0 OR	92	92	10
		
		3.0 OR	95	95	5
	
	60%	1.5 OR	58	90	92
		
		2.0 OR	75	99	99
		
		3.0 OR	73	93	94
	
	80%	1.5 OR	54	88	87
		
		2.0 OR	72	93	96
		
		3.0 OR	84	94	93
	
	95%	1.5 OR	0	92	92
		
		2.0 OR	0	83	83
		
		3.0 OR	0	93	93

The exact sensitivity, signal sensitivity and sensitivity with functional SNP removed, for MDR to identify a disease signal in data with 40% to 95% LD under two one-locus scenarios.

When the functional SNP was removed prior to MDR analysis, the results changed primarily for the models in populations with lower amounts of LD. With this functional SNP removed, it was no longer possible to measure the exact sensitivity and so only the signal sensitivity was examined. For the 40% LD situation, the mean sensitivity was 7 and 9.7 respectively when the SNP was located at the edge and middle of an LD block. In the 60% datasets, signal sensitivities of 62.3 and 95 were observed for detecting a SNP at the edge and middle of a block respectively. When the functional SNP was removed in 80% LD and 95% LD instances, the signal sensitivities remained the same as with the susceptibility locus in place with few small exceptions. With the functional locus dropped before analysis, the reason for decreased sensitivity in low LD models is due mostly to detection of SNPs with LD to the functional SNP below the 0.90 D' threshold.

### Analysis of Two-Locus Models

**Figure 5:**
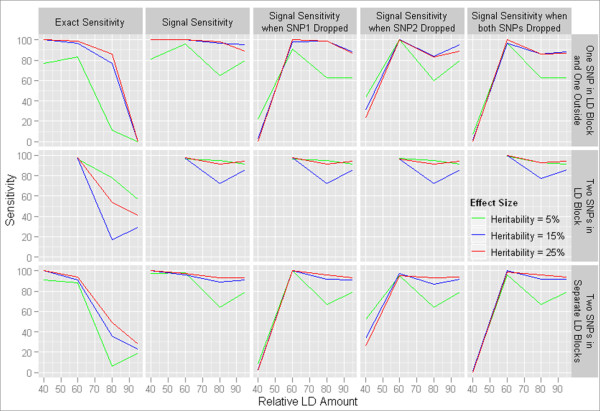
**Sensitivity of MDR for two-locus disease models**. The detection sensitivity of MDR when analyzing data with 40% LD, 60% LD, 80% LD or 95% LD and attempting to identify purely epistatic two-locus models.

Three types of two-locus epistatic interactions were examined: two SNPs in different blocks of LD, two SNPs in the same block of LD, two SNPs with one SNP inside an LD block and the other outside of any called block of LD. The instance in which two SNPs were in the same block of LD was not possible to simulate from the 40% LD data pool due to the lack of sufficiently large LD blocks. For each model, effect sizes of 5%, 10% and 15% broad-sense heritability were simulated. Each disease model was purely epistatic, meaning that there should be no detectable marginal effect from either functional SNP. For all two-locus models, analysis was run as described followed by a progression of removing one, the other, and finally both functional SNPs. The goal of this series of experiments is to determine whether or not MDR can detect the underlying genetic signal using LD (i.e. indirect association), or if it is limited in sensitivity to do so. The results of these MDR analyses are shown in Table 2 and trends in the data are illustrated in Figure 5. The case in which two functional SNPs were separated in two different LD blocks and the case where only one functional SNP resided in an LD block displayed similar trends. First, the sensitivity of MDR for most models increased proportionally with effect size from 5% to 15% heritability but not significantly between 15% and 25% heritability. MDR had high signal sensitivity in both low and high LD while exact sensitivity dropped in high LD datasets with two loci in separate LD blocks or only one locus in an LD block. In addition, the ability to detect the disease signals in absence of the actual functional SNPs increased with the amount of LD in the dataset. Surprisingly, when the SNP not in an LD block (SNP1) was dropped from high LD datasets before analysis, there was still considerable signal sensitivity. This phenomenon is most likely the result of patterns of long-range LD (not necessarily considered part of an LD block). With the exception of the models with lowest LD amounts, there was little difference in the signal sensitivities between analyses with one or both functional SNPs removed. Interestingly in some instances, there was more sensitivity with both SNPs removed than with the removal of only the second SNP. Once again, in many cases the drop in signal sensitivity with functional SNPs dropped results from selection of SNPs in LD below the threshold with the functional SNPs.

**Table 2:** Sensitivity of MDR to detect two-locus disease models

Scenario	Relative LD	Broad-Sense Heritability	Exact Sensitivity	Signal Sensitivity	Signal Sensitivity when SNP1 Dropped	Signal Sensitivity when SNP2 Dropped	Signal Sensitivity when both SNPs Dropped
One SNP in LD Block and One Outside	40%	5%	77	81	22	44	7
		
		15%	100	100	3	31	1
		
		25%	100	100	0	23	0
	
	60%	5%	83	96	91	100	97
		
		15%	97	100	98	100	97
		
		25%	99	100	100	100	100
	
	80%	5%	11	65	63	60	63
		
		15%	77	97	99	84	86
		
		25%	86	98	99	83	86
	
	95%	5%	0	80	63	80	63
		
		15%	0	95	88	95	88
		
		25%	0	89	87	89	87

Two SNPs in Separa te LD Blocks	40%	5%	91	97	8	52	1
		
		15%	100	100	3	34	1
		
		25%	100	100	2	26	0
	
	60%	5%	88	98	100	95	96
		
		15%	91	96	100	97	100
		
		25%	94	97	100	95	99
	
	80%	5%	6	64	67	64	67
		
		15%	36	89	92	87	92
		
		25%	49	93	96	93	96
	
	95%	5%	19	79	79	79	79
		
		15%	23	91	91	92	92
		
		25%	28	93	93	94	94

Two SNPs in Same LD Block	60%	5%	96	96	96	97	99
		
		15%	97	97	98	96	100
		
		25%	98	98	98	96	100
	
	80%	5%	78	95	95	95	93
		
		15%	17	72	72	72	77
		
		25%	54	91	91	91	93
	
	95%	5%	57	91	91	91	91
		
		15%	29	86	86	86	86
		
		25%	41	94	94	94	94

Detection sensitivity of MDR to identify functional SNPs from 40% to 95% LD amounts in epistatic two-locus models.

MDR analysis of epistatic models in which both functional loci were in the same block of LD yielded significantly different results and lead to a change in the constraints for measuring sensitivity, specifically for this type of model. Even though the two-locus interactions simulated were purely epistatic and had no main effects, MDR chose mostly one-locus models as the most accurate predictors. The signal sensitivity and exact sensitivity were almost always zero as a result (MDR failed to identify the exact two-locus model or two-locus model from the same LD block). We modified the constraints so that these one-locus choices were considered a positive signal towards exact sensitivity if they contained one of the functional loci and signal sensitivity if they were in LD with one of the functional SNPs with a D' of greater than or equal to 0.90; so MDR was not required to simultaneously detect both functional loci in the model. Models in which a false positive SNP was included in the model were not counted towards detection sensitivity, however. With this taken into account, the trends observed in the data are similar to those described for the other two model types. The signal sensitivity of MDR was high in all levels of LD while the exact sensitivity decreased with increasing LD as was seen before. For the most part, no increase in detection sensitivity was experienced with higher heritability. The detection sensitivity for all levels of LD was lower for 15% heritability than for 5% and 25%, which results from the more frequent selection of two-locus models with a false positive result in these 15% heritability results. The mean signal sensitivity based on all effect sizes ranged from 97 in 60% LD to 86 and 90.3 in 80% and 95% LD respectively. The exact sensitivity, however, decreased from 97 in 60% LD to 49.7 and 42.3 in 80% and 95% LD. Removing one of the functional SNPs before analysis did not strongly impact sensitivity.

## Discussion

Linkage disequilibrium (LD) often plays a significant role in guiding both the design and analysis of genome-wide association studies (GWAS). It is an implicit assumption of the GWAS that LD exists between a SNP typed in the genotyping assay and the functional variant causing disease. As a result, GWAS benefits from strong amounts of LD distributed throughout the genome. On the other hand, excessive LD can lead to the detection of statistical associations over large genomic regions and preclude easily narrowing the region surrounding a susceptibility locus. The presence of LD has been shown to be beneficial to the detection capabilities of certain data mining methods such as grammatical evolution neural networks [9]. This follows logically from the need to find signal peaks when not all potential models are tested. As MDR tests all possible N-way interaction models and does not rely on signal peaks to guide the search, the circumstances induced by LD are not equivalent. We have found that there are general trends associated with increasing strength of LD when analyzing data with MDR. These trends extend to the specific type of model being analyzed and although the type of model cannot be discerned before analyzing non-simulated data, it is important to be cognizant of these trends.

Firstly, the detection ability of MDR to identify the correct functional loci decreases proportionally to the amount of LD surrounding that locus. As the correlation between the disease susceptibility loci and SNPs surrounding it increases, the surrounding SNPs are able to predict disease status as well as the functional loci; thus there is equal probability that any of these loci will be selected by MDR. The result is that the embedded functional loci are not chosen as many times out of the one hundred datasets. This phenomenon also explains why higher sensitivity for detecting the disease loci when they are not directly genotyped is achieved as LD increases. High amounts of LD around causative loci result in SNPs that effectively tag the functional loci and allow the signal to be detected. This shows that MDR performs well when detecting the indirect association expected in a GWAS.

It was initially surprising to observe that MDR would use a one-locus model to predict disease status in some cases where the underlying etiology was a two-locus epistatic interaction. It made sense, however, that this phenomenon was only observed in instances of extensive LD. In such a case, the two functional SNPs were so highly correlated that the epistatic multi-locus interaction presents as a single-locus effect during analysis. The result is that only the genotype at one of the two loci is necessary to accurately predict disease status. Although it seems unlikely that two functional loci would be so highly correlated in practice, this is an interesting scenario to consider.

## Conclusions

**Figure 6:**
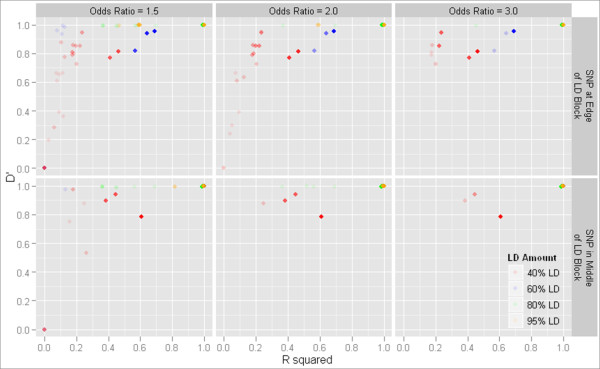
**D' and r^2 ^for one-locus models chosen by MDR**. D' and r^2 ^between models selected by MDR and the functional locus in cases where MDR picked a one-locus model. Points with no transparency indicate a count of at least 20 models.

**Figure 7:**
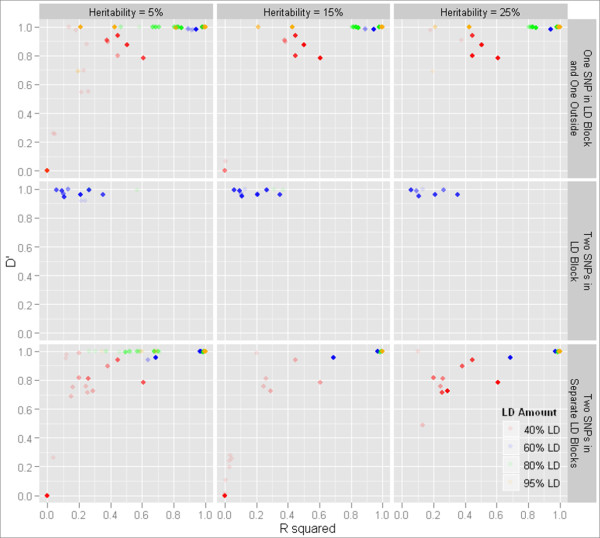
**D' and r^2 ^for two-locus models chosen by MDR**. D' and r^2 ^between each locus in models selected by MDR and each of the functional loci in cases where MDR picked a two-locus model. Solid points indicate a count of at least 20 models.

In drawing conclusions from the research presented in this paper, we wish to make recommendations about the future use of MDR in performing gene-gene interaction analysis in data with significant amounts of LD among the SNPs. We propose that the linkage disequilibrium structure surrounding MDR results should be carefully considered before undertaking a follow-up study. This includes both patterns of D' and r^2^. It has been discovered that MDR will sometimes select SNPs which possess a high D' with the functional SNP even if the r^2 ^would not be considered to be of significant strength (Figure 6, 7). Figure 6 shows the r^2 ^and D' of the single embedded disease SNP with all single-locus best models chosen during MDR analysis. Figure 7 displays the r^2 ^and D' of both functional SNPs participating in epistatic interactions with each of the SNPs selected by MDR only when the best model chosen was a two-locus model. SNPs selected with disproportionate D' and r^2 ^values tend to have a minor allele frequency (MAF) within 0.1 of the functional locus but the low resulting r^2 ^might preclude the follow-up of the functional locus these SNPs are tagging. This scenario is seen much more often as the size of the genetic effect increases. In such a case, MDR has a higher probability of selecting tagging SNPs with lower r^2 ^LD values. We recommend as a result that it might be wise to consider the area around the significant SNPs found which have high D' as well as those areas with high r^2^, at least for the purposes of replication. In addition, it might be useful to pick a tagging SNP for regions with extremely high levels of LD (r^2 ^> 0.90) when performing gene-gene interaction analysis with MDR, as this high level of LD could result in different best models being chosen in separate cross-validation intervals during the MDR process. This would in turn lower the cross-validation consistency of each of the models containing loci tagging the disease signal and reduce the detection sensitivity from MDR. While the current large-scale genetic association studies benefit from segments of linkage disequilibrium across the genome, it is important during quality control and analysis to consider possible detrimental effects of this disequilibrium.

## Methods

### The genomeSIMLA Simulation Process

The genomeSIMLA data simulation package is a forward-time genomic simulator that allows for the creation of genomic data with realistic patterns of LD [10,11]. The simulator works by first creating a definition for the layout of the chromosome with a template. It is from this chromosome template whereby specific instantiations will be drawn to create the population. The chromosomes used in genomeSIMLA consist of blocks of biallelic SNPs and are defined by the user, with variables such as the number of blocks, the number of variants within each block, and the recombination frequency within and between such blocks. In this way, a user can establish the number of SNPs in a chromosome as well as how often crossovers occur between SNPs. A population of chromosomes is created by initializing chromosomes with randomized values within the parameter specifications of the template chromosome. Subsequent to initialization, the population of chromosomes is advanced through a user-defined number of generations (usually 1000-1500) with population data being dropped at specified points along the way and changes to the population occurring based on the growth and recombination functions. Finally, a penetrance table is applied while drawing chromosomes from the one of the population-data drops to create case-control datasets. Two chromosomes are drawn from the population to determine each individual's genetic makeup and designations of case or control are given based on the disease probability function in the penetrance table. The drawing process continues until the required number of cases and controls are produced.

### Data Simulation

In order to make data pools with varying amounts of LD, genomeSIMLA was recursively run with varying recombination frequency and growth parameters. These recursive runs varied the growth rate of the Richards' growth curve [12] between 0.02 and 0.04 and the generation of maximum growth between 250 and 900. Recombination rate within a block of SNPs was also modified between 1 × 10^-7 ^and 1 × 10^-4^. The result was a group of 60 data pools, where each pool represents a population of simulated chromosomes. Each chromosome simulated in this study contained approximately 600 SNPs. Data pools representing 40%, 60%, 80%, and 95% relative LD (heretofore referred to as 40% LD, 60% LD, 80% LD and 95% LD) were selected for use in an MDR sensitivity analysis (Figure 2). Relative LD, in this case, is the ratio of SNPs in LD with at least one other SNP to the total number of polymorphic SNPs. All definitions of LD blocks utilized the block-calling algorithm innate to the genomeSIMLA software. SNPs in these representative data pools were chosen on the basis of meeting requirements of specified allele frequency and the following LD structures (as shown in Figure 3):

• One-locus with the functional SNP at the beginning of an LD block

• One-locus with the functional SNP in the middle of an LD block

• Two-locus with one functional SNP in a block of LD and the other outside of a called block

• Two-locus with both functional SNPs in separate blocks of LD

• Two-locus with both functional SNPs in the same LD block (N/A for 40% relative LD)

Penetrance functions with effect sizes of 5, 15 and 25% broad-sense heritability were applied to two-locus models while additive functions with per-allele odds ratios of 1.5, 2.0 and 3.0 were applied to one-locus models. Based upon the probability of disease at single- and multi-locus genotypes of the chosen functional SNPs given by the penetrance functions applied, 100 datasets containing 1000 cases and 1000 controls were drawn for each disease model and each scenario. To then assess MDR's potential to detect the signal of a functional SNP through LD, the functional SNP was removed from one-locus model datasets and both SNPs were removed in turn, and then together, from the two-locus disease datasets. Removal of functional loci was conducted subsequent to application of the penetrance function and prior to analysis. This can be thought of as simulating an indirect association. In total 18,000 datasets were simulated, including those which were generated by removing functional SNPs.

### Data Analysis

Multifactor Dimensionality Reduction (MDR) [3] was used to analyze simulated data. MDR utilizes a 4-step algorithm designed to detect gene-gene or gene-environment interactions by reducing the dimensionality of the interaction space. In step 1, a set of *n *genetic and/or discrete environmental factors is selected from the pool of all factors. In step 2, the *n *factors and their possible multifactor classes or cells are represented in *n*-dimensional space; for example, in the case of two biallelic markers with three possible genotypes, there are nine two-locus-genotype combinations. The ratio of the number of cases to the number of controls is estimated for each multifactor cell. In step 3, each multifactor cell in *n*-dimensional space is labeled either as "high-risk" if the case-control ratio meets or exceeds some threshold - usually the case-control ratio for the combined study population - or as "low-risk" if that threshold is not exceeded. In this way, a model for both cases and controls (or for affected and unaffected sibs) is formed by pooling high-risk cells into one group and low-risk cells into another group. This reduces the *n*-dimensional model to a one-dimensional model (i.e., having one variable with two multifactor classes--high risk and low risk). In step 4, the prediction error of each model is estimated by N-fold cross-validation. Here, the data (i.e., subjects) are randomly divided into N equal parts. The MDR model is iteratively developed for each possible N-1 partitions of the subjects and then is used to make predictions about the disease status for the partition of the subjects excluded. The proportion of subjects for which an incorrect prediction was made is an estimation of the prediction error.

Our simulated data was analyzed with MDR using 10-fold cross-validation exhaustively analyzing all one- and two-locus models. The resulting models were ranked by cross-validation consistency and balanced accuracy - a metric of the classification and prediction accuracy averaged over all cross-validation intervals - and the single best model was chosen. The exact sensitivity of MDR was judged by the number of times out of the 100 datasets in which the best model contained all embedded functional loci. In addition, the signal sensitivity was determined by the number of times which MDR chose a best model in which the loci were in D' greater than or equal to 0.90 with the functional loci. The signal sensitivity refers to the ability of MDR to detect the disease signal, even if the algorithm was unable to pick up the exact functional loci.

## Competing interests

The authors declare that they have no competing interests.

## Authors' contributions

BJG designed the study, carried out simulations, performed MDR analysis and drafted the manuscript. EST scripted the parallelization of simulations and MDR analyses. MDR assisted with study design and helped in the drafting of the manuscript. All authors read and approved the final manuscript.
